# Conformation and absolute configuration of (1*S*,2*S*)-2-(phenyl­selan­yl)cyclo­hexyl (*R*)-2-meth­oxy-2-(1-naphth­yl)propionate

**DOI:** 10.1107/S1600536810022233

**Published:** 2010-06-16

**Authors:** Satoshi Murakami, Akane Kato, Hiroshi Katagiri, Takatoshi Matsumoto, Tatsuro Kijima

**Affiliations:** a Department of Biochemical Engineering, Graduate School of Science and Engineering, Yamagata University, 4-3-16 Jonan, Yonezawa, Yamagata 992-8510, Japan; b Institute of Multidisciplinary Research for Advanced Materials, Tohoku University, 2-2-1 Katahira, Aoba, Sendai 980-8577, Japan

## Abstract

The relative and absolute configurations of the title compound, C_26_H_28_O_3_Se, were assigned from the known configuration of (*R*)-(−)-2-meth­oxy-2-(1-naphth­yl)propionic acid used as starting material, and by examination of the Bijvoet (Friedel) pairs, using the anomalous dispersion data collected with Mo *K*α radiation at low temperature. The geometry around the carbonyl group exists in the *syn* conformation, as reflected in torsion angles involving this group, and the stability of the structure is affected by weak bifurcated intra­molecular C—H⋯O hydrogen bonds.

## Related literature

For general background to the crystalline-state analysis of 2-meth­oxy-2-(1-naphth­yl)propionic acid ester, see: Kuwahara *et al.* (2007 ▶). For synthetic details, see: Detty (1980 ▶); Izumi *et al.* (1993 ▶); Harada *et al.* (2000 ▶). For Bijvoet pairs analysis, see: Hooft *et al.* (2008 ▶).
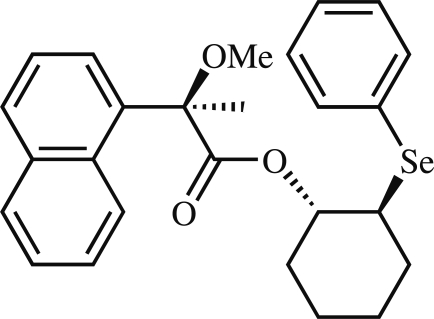


## Experimental

### 

#### Crystal data

C_26_H_28_O_3_Se
*M* *_r_* = 467.44
Orthorhombic, 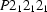

*a* = 7.5714 (3) Å
*b* = 15.9740 (7) Å
*c* = 18.2994 (8) Å
*V* = 2213.23 (16) Å^3^
*Z* = 4
Mo *K*α radiation
μ = 1.72 mm^−1^
*T* = 115 K
0.30 × 0.10 × 0.08 mm

#### Data collection

Rigaku R-AXIS RAPID diffractometer
Absorption correction: multi-scan (*ABSCOR*; Higashi, 1995 ▶) *T*_min_ = 0.827, *T*_max_ = 1.000
21509 measured reflections
5060 independent reflections
4749 reflections with *I* > 2σ(*I*)
*R*_int_ = 0.032

#### Refinement

*R*[*F* ^2^ > 2σ(*F* ^2^)] = 0.024
*wR*(*F* ^2^) = 0.055
*S* = 1.06
5060 reflections
274 parameters
H-atom parameters constrained
Δρ_max_ = 0.47 e Å^−3^
Δρ_min_ = −0.31 e Å^−3^
Absolute structure: Flack (1983 ▶), 2178 Friedel pairs
Flack parameter: −0.015 (6)

### 

Data collection: *PROCESS-AUTO* (Rigaku, 1998 ▶); cell refinement: *PROCESS-AUTO*; data reduction: *CrystalStructure* (Rigaku/MSC, 2003 ▶); program(s) used to solve structure: *SHELXS97* (Sheldrick, 2008 ▶); program(s) used to refine structure: *SHELXL97* (Sheldrick, 2008 ▶); molecular graphics: *PLATON* (Spek, 2009 ▶); software used to prepare material for publication: *Yadokari-XG* (Wakita, 2001 ▶; Kabuto *et al.*, 2009 ▶).

## Supplementary Material

Crystal structure: contains datablocks I, global. DOI: 10.1107/S1600536810022233/bh2291sup1.cif

Structure factors: contains datablocks I. DOI: 10.1107/S1600536810022233/bh2291Isup2.hkl

Additional supplementary materials:  crystallographic information; 3D view; checkCIF report

## Figures and Tables

**Table 1:** Selected torsion angles (°)

O1—C1—C3—O2	−17.1 (2)
O2—C3—O3—C14	−2.8 (2)
C1—C3—O3—C14	174.70 (13)
H14—C14—O3—C3	−27

**Table 2:** Geometry of the weak bifurcated intra­molecular C—H⋯O hydrogen bonds (Å, °)

*D*—H⋯*A*	*D*—H	H⋯*A*	*D*⋯*A*	*D*—H⋯*A*
C12—H12⋯O1	0.95	2.42	3.000 (1)	119
C12—H12⋯O2	0.95	2.65	3.405 (2)	137

**Fig. 1.**
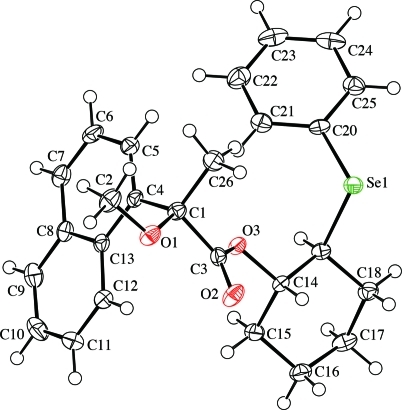
The asymmetric unit of the title compound, showing 50% probability displacement ellipsoids. H atoms are shown as spheres.

**Fig. 2.**
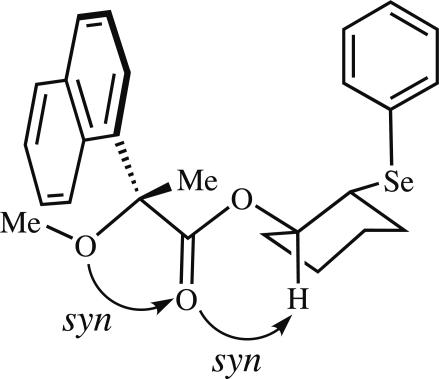
The preferred conformation of (R)-MαNP acid ester, adopting syn/syn conformation (i.e., the so-called syn conformation).

**Fig. 3.**
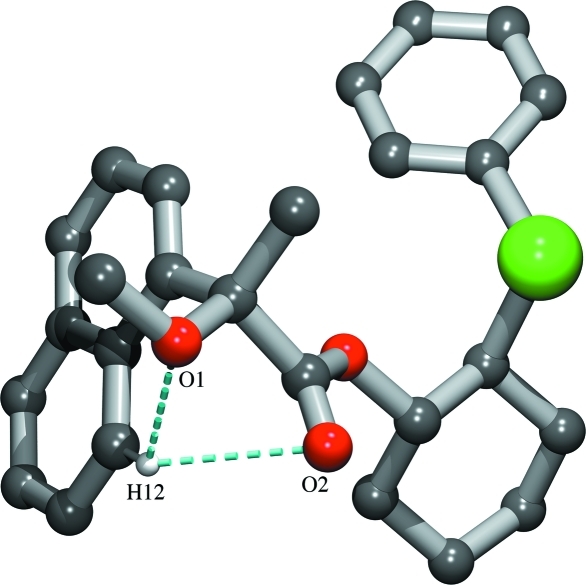
The weak bifurcated intramolecular C—H···O hydrogen bonds, stabilizing the syn conformation.

**Fig. 4.**
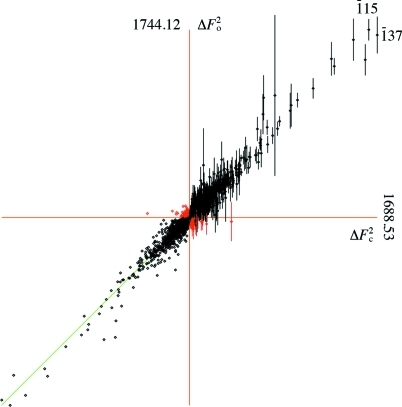
A scatter plot of Bijvoet differences, prepared using the program PLATON (Spek, 2009). Shown are 1927 pairs where Δobs > 0.25σ(Δobs). 1750 reflections confirming the absolute structure are shown in black. 177 reflections with the wrong sign are shown in red.

## supplementary crystallographic information

### Comment

Previously, Izumi group presented a chemoenzymatic synthesis of optically pure (*R*)- and (*S*)-2-cyclohexen-1-ols (Izumi *et al.*, 1993). However, the preparation and determination of the absolute configurations of such kind of aliphatic alcohols still has been difficult and important topic. The *M*αNP [2-methoxy-2-(1-naphthyl)- propionic] acid method is an attractive approach for the preparation of enantiopure alcohols and the determination of their absolute configurations by ^1^H NMR anisotropy or X-ray crystallography (Harada *et al.*, 2000). Recent systematic X-ray crystallographic analysis of *M*αNP acid esters with various alcohols has shown that most prefer the *syn*/*syn* conformation (the so-called "*syn*" conformation) (Kuwahara *et al.*, 2007). On the other hand, although selenium has a large *f"*-value (Δ*f"*=2.223 for Mo *K*α radiation), which promises a large anomalous scattering effect, *M*αNP acid esters including a Se atom have not been investigated by X-ray analysis. The structural properties and determination of the absolute configuration of such compounds are important for the development of this methodology.

We report here the conformation and absolute configuration of (*R*)-2-methoxy-2-(1-naphthyl)-propionic acid (1*S*,2*S*)-2-(phenylseleno)-cyclohexyl ester. Hydrolysis of this compound gives (+)-*trans*-2-(phenylseleno)-cyclohexan-1-ol, in which an olefin-forming *syn* elimination of phenyl selenoxide forms 2-cyclohexen-1-ol, whose optically active forms are especially useful in the asymmetric synthesis of terpenes and other natural products.

The crystal structure of the title compound (Fig. 1) showed the following torsion angles: O1—C1—C3—O2, -17.1 (2)°; O2—C3—O3—C14, -2.8 (2)°; C1—C3—O3—O14, 174.70 (13)°; and H14—C14—O3—C3, -27°. These angles indicate that the geometry around the carbonyl group takes the *syn* conformation (Table 1, Fig. 2). Moreover, weak bifurcated intramolecular hydrogen bonds in O1···H12···O2 showed a triangular shape (Table 2, Fig. 3). These structural properties are similar to those of most *M*αNP acid esters (Kuwahara *et al.*, 2007). The absolute structure was determined from the known configuration of *M*αNP, as supported by the refined Flack *χ* parameter and the Bijvoet pairs analysis of Hooft *et al.* (2008) performed with the *PLATON* program (Spek, 2009). The *P*2 parameter was 1.000 and the Hooft *y* parameter was -0.013. The plot of 1927 Bijvoet pairs in Fig. 4 suggests that the absolute configuration could be determined with a high confidence.

### Experimental

To a mixture of enantiopure (*R*)-(–)-*M*αNP acid (4.04 mmol), 4-dimethylaminopyridine (DMAP, 2.45 mmol), (±)-*trans*-2-(phenylseleno)-cyclohexan-1-ol (Detty, 1980) (2.96 mmol), and *N*,*N*'-diisopropyl-carbodiimide guanidine (DIC, 8.23 mmol) in CH_2_Cl_2_ (6.4 ml) cooled at 273 K was added 10-camphorsulfonic acid (CSA, 0.49 mmol), and the mixture was stirred at room temperature overnight. After addition of water (0.5 ml), the mixture was stirred for 1 h, diluted with EtOAc, and filtered with Celite, which was washed with EtOAc. The organic layer was evaporated under reduced pressure, and the residue was subjected to short column chromatography on silica gel (EtOAc). The crude diastereomeric esters obtained were separated by HPLC on silica gel (hexane/EtOAc = 30:1) giving the title compound as a second-eluted ester in 48.5% yield (colorless). [α]_D_^22^ 67.746 (c 1.42, CHCl_3_), mp = 405–406 K. Crystals suitable for X-ray diffraction were grown by slow solvent/solvent diffusion of hexane into ethyl acetate. IR (KBr): ν[cm^-1^] 2941, 1447 (alkane), 1739 (ester CO), 1136, 1055, 1022, 785 (mono substituted benzene) ^1^H NMR (400 MHz, CDCl_3_): *δ*[p.p.m.] 0.67–1.96 (m, 8H) 2.03 (s, 3H) 2.90(ddd, 1H, *J *= 10.07, 10.07 and 4.12 Hz) 3.07 (s, 3H) 4.78 (ddd, 1H, *J *= 9.62, 9.62 and 4.12 Hz) 7.22–7.30 (m, 1H) 7.44–7.49 (m, 5H) 7.57–7.59 (m, 3H) 7.83–7.87(m, 1H) 8.42–8.46 (m, 2H). ^13^C NMR (100 MHz, CDCl_3_): δ [p.p.m.] 21.5, 23.1, 25.4, 30.6, 32.5, 45.5, 50.8, 75.3, 81.4, 124.5, 125.4, 125.7, 125.9, 126.4, 127.5, 128.9, 129.4, 131.4, 134.0, 134.9, 135.0, 173.5 Anal. Calcd. for C_26_H_28_O_3_Se: C 66.8, H 5.99%. Found: C 66.71, H 6.08%.

### Refinement

In the refinement of the title compound, the H atoms were calculated geometrically and refined as riding, with C—H bond lengths of 0.95–1.00 Å, and with *U*_iso_(H) values of 1.2*U*_eq_(C) or 1.5*U*_eq_(methyl C).

### Crystal data

**Table:**

C_26_H_28_O_3_Se	*D*_x_ = 1.403 Mg m^−^^3^
*M**_r_* = 467.44	Melting point: 405 K
Orthorhombic, *P*2_1_2_1_2_1_	Mo *K*α radiation, λ = 0.71075 Å
Hall symbol: P 2ac 2ab	Cell parameters from 19734 reflections
*a* = 7.5714 (3) Å	θ = 3.2–27.5°
*b* = 15.9740 (7) Å	µ = 1.72 mm^−^^1^
*c* = 18.2994 (8) Å	*T* = 115 K
*V* = 2213.23 (16) Å^3^	Plate, colourless
*Z* = 4	0.30 × 0.10 × 0.08 mm
*F*(000) = 968	

### Data collection

**Table:**

Rigaku R-AXIS RAPID diffractometer	5060 independent reflections
Radiation source: rotating anode	4749 reflections with *I* > 2σ(*I*)
graphite	*R*_int_ = 0.032
ω scans	θ_max_ = 27.5°, θ_min_ = 3.2°
Absorption correction: multi-scan (*ABSCOR*; Higashi, 1995)	*h* = −8→9
*T*_min_ = 0.827, *T*_max_ = 1.000	*k* = −20→20
21509 measured reflections	*l* = −23→23

### Refinement

**Table:**

Refinement on *F*^2^	Hydrogen site location: inferred from neighbouring sites
Least-squares matrix: full	H-atom parameters constrained
*R*[*F*^2^ > 2σ(*F*^2^)] = 0.024	*w* = 1/[σ^2^(*F*_o_^2^) + (0.0284*P*)^2^ + 0.3422*P*] where *P* = (*F*_o_^2^ + 2*F*_c_^2^)/3
*wR*(*F*^2^) = 0.055	(Δ/σ)_max_ = 0.001
*S* = 1.06	Δρ_max_ = 0.47 e Å^−^^3^
5060 reflections	Δρ_min_ = −0.31 e Å^−^^3^
274 parameters	Extinction correction: *SHELXL97* (Sheldrick, 2008), Fc^*^=kFc[1+0.001xFc^2^λ^3^/sin(2θ)]^-1/4^
0 restraints	Extinction coefficient: 0.0023 (4)
0 constraints	Absolute structure: Flack (1983), 2178 Friedel pairs
Primary atom site location: structure-invariant direct methods	Flack parameter: −0.015 (6)
Secondary atom site location: difference Fourier map	

### Fractional atomic coordinates and isotropic or equivalent isotropic displacement parameters (Å^2^)

**Table:**

	*x*	*y*	*z*	*U*_iso_*/*U*_eq_	
C1	−0.1779 (2)	0.23902 (10)	0.24894 (9)	0.0192 (3)	
O1	−0.33156 (17)	0.20184 (7)	0.21696 (7)	0.0228 (3)	
C2	−0.4470 (2)	0.25838 (13)	0.18042 (10)	0.0287 (4)	
H2	−0.5034	0.2952	0.2163	0.043*	
H2A	−0.5378	0.2267	0.1542	0.043*	
H2B	−0.3792	0.2922	0.1456	0.043*	
C3	−0.0844 (2)	0.16503 (11)	0.28614 (9)	0.0186 (4)	
O2	−0.15312 (17)	0.09919 (8)	0.29975 (7)	0.0268 (3)	
O3	0.08202 (16)	0.18555 (8)	0.30378 (7)	0.0188 (3)	
C4	−0.0564 (2)	0.27792 (10)	0.19115 (9)	0.0174 (3)	
C5	−0.0072 (2)	0.36005 (11)	0.19467 (10)	0.0216 (4)	
H5	−0.0532	0.3939	0.2329	0.026*	
C6	0.1098 (3)	0.39644 (12)	0.14346 (11)	0.0243 (4)	
H6	0.1405	0.4539	0.1472	0.029*	
C7	0.1782 (3)	0.34865 (11)	0.08873 (10)	0.0236 (4)	
H7	0.2570	0.3730	0.0544	0.028*	
C8	0.1328 (2)	0.26292 (12)	0.08248 (9)	0.0204 (4)	
C9	0.2077 (2)	0.21244 (13)	0.02694 (10)	0.0264 (4)	
H9	0.2879	0.2367	−0.0069	0.032*	
C10	0.1665 (3)	0.12967 (13)	0.02130 (10)	0.0308 (4)	
H10	0.2197	0.0963	−0.0156	0.037*	
C11	0.0448 (3)	0.09373 (12)	0.07026 (11)	0.0286 (4)	
H11	0.0149	0.0362	0.0657	0.034*	
C12	−0.0308 (3)	0.14082 (11)	0.12443 (10)	0.0227 (4)	
H12	−0.1133	0.1155	0.1567	0.027*	
C13	0.0117 (2)	0.22675 (10)	0.13327 (9)	0.0181 (4)	
C14	0.1779 (3)	0.12243 (10)	0.34533 (9)	0.0174 (3)	
H14	0.0914	0.0880	0.3736	0.021*	
C15	0.2795 (3)	0.06561 (11)	0.29373 (10)	0.0241 (4)	
H15	0.3670	0.0988	0.2659	0.029*	
H15A	0.1972	0.0393	0.2585	0.029*	
C16	0.3739 (3)	−0.00205 (12)	0.33817 (11)	0.0259 (4)	
H16	0.2852	−0.0382	0.3624	0.031*	
H16A	0.4451	−0.0375	0.3050	0.031*	
C17	0.4935 (2)	0.03679 (11)	0.39562 (12)	0.0262 (4)	
H17	0.5916	0.0667	0.3712	0.031*	
H17A	0.5451	−0.0081	0.4262	0.031*	
C18	0.3924 (2)	0.09780 (11)	0.44410 (10)	0.0225 (4)	
H18	0.4750	0.1244	0.4790	0.027*	
H18A	0.3024	0.0668	0.4726	0.027*	
C19	0.3018 (2)	0.16562 (10)	0.39842 (10)	0.0190 (4)	
H19	0.3937	0.1966	0.3700	0.023*	
Se1	0.17856 (2)	0.245122 (11)	0.462912 (9)	0.02078 (6)	
C20	0.2786 (2)	0.34792 (11)	0.42811 (11)	0.0191 (4)	
C21	0.2687 (3)	0.37120 (12)	0.35488 (11)	0.0246 (4)	
H21	0.2171	0.3345	0.3201	0.030*	
C22	0.3347 (3)	0.44831 (12)	0.33294 (11)	0.0305 (4)	
H22	0.3282	0.4640	0.2829	0.037*	
C23	0.4096 (3)	0.50226 (12)	0.38291 (14)	0.0335 (5)	
H23	0.4554	0.5547	0.3674	0.040*	
C24	0.4177 (3)	0.47954 (12)	0.45598 (13)	0.0324 (5)	
H24	0.4683	0.5168	0.4906	0.039*	
C25	0.3522 (2)	0.40256 (12)	0.47872 (11)	0.0251 (4)	
H25	0.3577	0.3873	0.5288	0.030*	
C26	−0.2293 (2)	0.29874 (12)	0.31135 (11)	0.0251 (4)	
H26	−0.2946	0.3465	0.2914	0.038*	
H26A	−0.1223	0.3188	0.3359	0.038*	
H26B	−0.3038	0.2690	0.3466	0.038*	

### Atomic displacement parameters (Å^2^)

**Table:**

	*U*^11^	*U*^22^	*U*^33^	*U*^12^	*U*^13^	*U*^23^
C1	0.0175 (7)	0.0183 (8)	0.0218 (7)	0.0003 (8)	−0.0016 (7)	0.0013 (7)
O1	0.0186 (6)	0.0210 (6)	0.0289 (7)	−0.0014 (5)	−0.0043 (6)	0.0050 (5)
C2	0.0237 (8)	0.0292 (10)	0.0333 (10)	0.0021 (9)	−0.0057 (8)	0.0075 (9)
C3	0.0207 (9)	0.0206 (9)	0.0143 (8)	0.0009 (7)	0.0011 (7)	0.0005 (7)
O2	0.0241 (7)	0.0237 (6)	0.0325 (7)	−0.0049 (6)	−0.0031 (6)	0.0113 (6)
O3	0.0201 (6)	0.0158 (6)	0.0205 (6)	−0.0010 (5)	−0.0023 (5)	0.0041 (5)
C4	0.0175 (8)	0.0175 (8)	0.0172 (8)	0.0013 (6)	−0.0035 (7)	0.0027 (6)
C5	0.0244 (9)	0.0185 (8)	0.0220 (9)	0.0014 (7)	−0.0036 (7)	0.0006 (7)
C6	0.0274 (10)	0.0155 (8)	0.0301 (10)	−0.0036 (7)	−0.0042 (8)	0.0065 (8)
C7	0.0209 (8)	0.0261 (9)	0.0239 (9)	−0.0056 (8)	−0.0013 (8)	0.0068 (7)
C8	0.0187 (7)	0.0240 (9)	0.0184 (8)	0.0009 (7)	−0.0033 (6)	0.0032 (7)
C9	0.0225 (9)	0.0364 (10)	0.0202 (9)	−0.0006 (8)	0.0012 (8)	0.0004 (8)
C10	0.0321 (10)	0.0375 (11)	0.0227 (10)	0.0045 (9)	0.0012 (9)	−0.0100 (8)
C11	0.0377 (11)	0.0210 (9)	0.0271 (10)	0.0006 (9)	−0.0027 (9)	−0.0054 (8)
C12	0.0261 (9)	0.0201 (9)	0.0219 (9)	−0.0001 (7)	−0.0019 (8)	0.0012 (7)
C13	0.0184 (8)	0.0194 (9)	0.0164 (8)	0.0007 (6)	−0.0049 (6)	0.0017 (6)
C14	0.0208 (8)	0.0137 (7)	0.0176 (8)	0.0009 (8)	−0.0005 (8)	0.0029 (6)
C15	0.0333 (11)	0.0176 (8)	0.0214 (9)	0.0015 (7)	0.0042 (8)	−0.0011 (7)
C16	0.0321 (10)	0.0177 (9)	0.0278 (10)	0.0050 (8)	0.0063 (8)	0.0016 (8)
C17	0.0210 (9)	0.0188 (9)	0.0388 (11)	0.0035 (7)	0.0008 (8)	0.0032 (8)
C18	0.0216 (9)	0.0194 (9)	0.0266 (10)	0.0011 (7)	−0.0058 (8)	0.0002 (7)
C19	0.0180 (8)	0.0169 (8)	0.0220 (9)	0.0021 (7)	0.0002 (8)	−0.0001 (7)
Se1	0.02435 (9)	0.01706 (8)	0.02093 (8)	−0.00017 (8)	0.00258 (7)	−0.00066 (8)
C20	0.0170 (9)	0.0138 (8)	0.0266 (10)	0.0032 (6)	0.0016 (7)	−0.0002 (7)
C21	0.0245 (10)	0.0234 (9)	0.0259 (10)	0.0015 (7)	0.0009 (8)	−0.0025 (8)
C22	0.0319 (11)	0.0271 (9)	0.0326 (11)	0.0027 (9)	0.0080 (10)	0.0058 (8)
C23	0.0301 (10)	0.0173 (9)	0.0530 (14)	−0.0004 (8)	0.0083 (11)	0.0021 (9)
C24	0.0274 (10)	0.0197 (9)	0.0500 (14)	−0.0010 (7)	−0.0004 (10)	−0.0113 (9)
C25	0.0232 (9)	0.0230 (9)	0.0289 (11)	0.0030 (7)	−0.0027 (8)	−0.0043 (7)
C26	0.0246 (9)	0.0261 (10)	0.0246 (10)	0.0028 (7)	0.0032 (8)	−0.0010 (8)

### Geometric parameters (Å, °)

**Table:**

C1—O1	1.431 (2)	C14—H14	1.0000
C1—C4	1.533 (2)	C15—C16	1.530 (3)
C1—C3	1.537 (2)	C15—H15	0.9900
C1—C26	1.538 (2)	C15—H15A	0.9900
O1—C2	1.424 (2)	C16—C17	1.520 (3)
C2—H2	0.9800	C16—H16	0.9900
C2—H2A	0.9800	C16—H16A	0.9900
C2—H2B	0.9800	C17—C18	1.524 (3)
C3—O2	1.199 (2)	C17—H17	0.9900
C3—O3	1.341 (2)	C17—H17A	0.9900
O3—C14	1.457 (2)	C18—C19	1.531 (2)
C4—C5	1.365 (2)	C18—H18	0.9900
C4—C13	1.434 (2)	C18—H18A	0.9900
C5—C6	1.415 (3)	C19—Se1	1.9688 (17)
C5—H5	0.9500	C19—H19	1.0000
C6—C7	1.362 (3)	Se1—C20	1.9172 (18)
C6—H6	0.9500	C20—C25	1.389 (3)
C7—C8	1.416 (2)	C20—C21	1.393 (3)
C7—H7	0.9500	C21—C22	1.389 (3)
C8—C9	1.416 (3)	C21—H21	0.9500
C8—C13	1.428 (2)	C22—C23	1.378 (3)
C9—C10	1.362 (3)	C22—H22	0.9500
C9—H9	0.9500	C23—C24	1.387 (3)
C10—C11	1.407 (3)	C23—H23	0.9500
C10—H10	0.9500	C24—C25	1.390 (3)
C11—C12	1.370 (3)	C24—H24	0.9500
C11—H11	0.9500	C25—H25	0.9500
C12—C13	1.419 (2)	C26—H26	0.9800
C12—H12	0.9500	C26—H26A	0.9800
C14—C19	1.516 (2)	C26—H26B	0.9800
C14—C15	1.519 (2)		
			
O1—C1—C4	111.94 (13)	C14—C15—H15	109.8
O1—C1—C3	103.67 (13)	C16—C15—H15	109.8
C4—C1—C3	109.94 (13)	C14—C15—H15A	109.8
O1—C1—C26	110.80 (13)	C16—C15—H15A	109.8
C4—C1—C26	114.38 (14)	H15—C15—H15A	108.3
C3—C1—C26	105.35 (14)	C17—C16—C15	110.95 (15)
C2—O1—C1	115.32 (13)	C17—C16—H16	109.4
O1—C2—H2	109.5	C15—C16—H16	109.4
O1—C2—H2A	109.5	C17—C16—H16A	109.4
H2—C2—H2A	109.5	C15—C16—H16A	109.4
O1—C2—H2B	109.5	H16—C16—H16A	108.0
H2—C2—H2B	109.5	C16—C17—C18	111.37 (15)
H2A—C2—H2B	109.5	C16—C17—H17	109.4
O2—C3—O3	124.87 (16)	C18—C17—H17	109.4
O2—C3—C1	124.52 (16)	C16—C17—H17A	109.4
O3—C3—C1	110.56 (14)	C18—C17—H17A	109.4
C3—O3—C14	115.12 (13)	H17—C17—H17A	108.0
C5—C4—C13	118.98 (16)	C17—C18—C19	111.08 (16)
C5—C4—C1	121.36 (16)	C17—C18—H18	109.4
C13—C4—C1	119.63 (15)	C19—C18—H18	109.4
C4—C5—C6	122.31 (18)	C17—C18—H18A	109.4
C4—C5—H5	118.8	C19—C18—H18A	109.4
C6—C5—H5	118.8	H18—C18—H18A	108.0
C7—C6—C5	119.67 (17)	C14—C19—C18	107.76 (14)
C7—C6—H6	120.2	C14—C19—Se1	112.61 (12)
C5—C6—H6	120.2	C18—C19—Se1	109.97 (12)
C6—C7—C8	120.62 (17)	C14—C19—H19	108.8
C6—C7—H7	119.7	C18—C19—H19	108.8
C8—C7—H7	119.7	Se1—C19—H19	108.8
C9—C8—C7	120.77 (16)	C20—Se1—C19	99.56 (8)
C9—C8—C13	119.60 (17)	C25—C20—C21	119.68 (18)
C7—C8—C13	119.63 (17)	C25—C20—Se1	118.37 (15)
C10—C9—C8	121.02 (18)	C21—C20—Se1	121.81 (14)
C10—C9—H9	119.5	C22—C21—C20	119.72 (19)
C8—C9—H9	119.5	C22—C21—H21	120.1
C9—C10—C11	119.84 (18)	C20—C21—H21	120.1
C9—C10—H10	120.1	C23—C22—C21	120.7 (2)
C11—C10—H10	120.1	C23—C22—H22	119.6
C12—C11—C10	120.69 (18)	C21—C22—H22	119.6
C12—C11—H11	119.7	C22—C23—C24	119.61 (19)
C10—C11—H11	119.7	C22—C23—H23	120.2
C11—C12—C13	121.24 (18)	C24—C23—H23	120.2
C11—C12—H12	119.4	C23—C24—C25	120.29 (19)
C13—C12—H12	119.4	C23—C24—H24	119.9
C12—C13—C8	117.59 (16)	C25—C24—H24	119.9
C12—C13—C4	123.63 (16)	C20—C25—C24	119.97 (19)
C8—C13—C4	118.76 (16)	C20—C25—H25	120.0
O3—C14—C19	109.13 (13)	C24—C25—H25	120.0
O3—C14—C15	109.97 (14)	C1—C26—H26	109.5
C19—C14—C15	110.91 (16)	C1—C26—H26A	109.5
O3—C14—H14	108.9	H26—C26—H26A	109.5
C19—C14—H14	108.9	C1—C26—H26B	109.5
C15—C14—H14	108.9	H26—C26—H26B	109.5
C14—C15—C16	109.16 (15)	H26A—C26—H26B	109.5
			
C4—C1—O1—C2	−63.60 (18)	C9—C8—C13—C4	177.18 (15)
C3—C1—O1—C2	177.95 (14)	C7—C8—C13—C4	−2.1 (2)
C26—C1—O1—C2	65.38 (19)	C5—C4—C13—C12	−179.93 (17)
O1—C1—C3—O2	−17.1 (2)	C1—C4—C13—C12	1.9 (2)
C4—C1—C3—O2	−136.89 (18)	C5—C4—C13—C8	1.8 (2)
C26—C1—C3—O2	99.4 (2)	C1—C4—C13—C8	−176.35 (14)
O1—C1—C3—O3	165.38 (13)	C3—O3—C14—C19	−145.77 (15)
C4—C1—C3—O3	45.56 (18)	C3—O3—C14—C15	92.36 (17)
C26—C1—C3—O3	−78.15 (17)	O3—C14—C15—C16	−178.00 (14)
O2—C3—O3—C14	−2.8 (2)	C19—C14—C15—C16	61.19 (19)
C1—C3—O3—C14	174.70 (13)	C14—C15—C16—C17	−56.3 (2)
O1—C1—C4—C5	124.21 (17)	C15—C16—C17—C18	54.3 (2)
C3—C1—C4—C5	−121.13 (18)	C16—C17—C18—C19	−55.9 (2)
C26—C1—C4—C5	−2.9 (2)	O3—C14—C19—C18	176.81 (14)
O1—C1—C4—C13	−57.72 (19)	C15—C14—C19—C18	−61.88 (19)
C3—C1—C4—C13	56.94 (19)	O3—C14—C19—Se1	55.36 (16)
C26—C1—C4—C13	175.21 (15)	C15—C14—C19—Se1	176.66 (11)
C13—C4—C5—C6	−0.4 (3)	C17—C18—C19—C14	58.60 (19)
C1—C4—C5—C6	177.64 (16)	C17—C18—C19—Se1	−178.31 (12)
C4—C5—C6—C7	−0.5 (3)	C14—C19—Se1—C20	−113.12 (12)
C5—C6—C7—C8	0.2 (3)	C18—C19—Se1—C20	126.70 (13)
C6—C7—C8—C9	−178.12 (17)	C19—Se1—C20—C25	−128.14 (14)
C6—C7—C8—C13	1.2 (3)	C19—Se1—C20—C21	56.15 (16)
C7—C8—C9—C10	179.07 (18)	C25—C20—C21—C22	0.9 (3)
C13—C8—C9—C10	−0.2 (3)	Se1—C20—C21—C22	176.54 (15)
C8—C9—C10—C11	1.4 (3)	C20—C21—C22—C23	−0.2 (3)
C9—C10—C11—C12	−1.0 (3)	C21—C22—C23—C24	−0.5 (3)
C10—C11—C12—C13	−0.5 (3)	C22—C23—C24—C25	0.5 (3)
C11—C12—C13—C8	1.6 (3)	C21—C20—C25—C24	−0.9 (3)
C11—C12—C13—C4	−176.73 (17)	Se1—C20—C25—C24	−176.69 (14)
C9—C8—C13—C12	−1.2 (2)	C23—C24—C25—C20	0.2 (3)
C7—C8—C13—C12	179.46 (16)	H14—C14—O3—C3	−27

### Hydrogen-bond geometry (Å, °)

**Table:**

*D*—H···*A*	*D*—H	H···*A*	*D*···*A*	*D*—H···*A*
C6—H6···O2^i^	0.95	2.52	3.417 (2)	158
C12—H12···O1	0.95	2.42	3.001 (2)	119
C14—H14···O2	1.00	2.30	2.667 (3)	100
C17—H17···O2^ii^	0.99	2.39	3.351 (2)	163
C18—H18···Se1^iii^	0.99	2.80	3.7264 (17)	156

Symmetry codes: (i) −*x*, *y*+1/2, −*z*+1/2; (ii) *x*+1, *y*, *z*; (iii) *x*+1/2, −*y*+1/2, −*z*+1.

### Table 2 Geometry of the weak bifurcated intramolecular C—H···O hydrogen bonds (Å, °)

**Table:**

D—H···A	D—H	H···A	D···A	D—H···A
C12—H12···O1	0.95	2.42	3.000 (1)	119
C12—H12···O2	0.95	2.65	3.405 (2)	137
